# Impact of supradiaphragmatic lymphadenectomy on the survival of patients in stage IVB ovarian cancer with thoracic lymph node metastasis

**DOI:** 10.3389/fonc.2023.1203127

**Published:** 2023-08-10

**Authors:** Soo Jin Park, Kwon Joong Na, Maria Lee, In Kyu Park, Hyun Hoon Chung, Chang Hyun Kang, Jae-Weon Kim, Noh Hyun Park, Young-Tae Kim, Yong Sang Song, Samina Park, Hee Seung Kim

**Affiliations:** ^1^ Department of Obstetrics and Gynecology, Seoul National University Hospital, Seoul, Republic of Korea; ^2^ Department of Thoracic and Cardiovascular Surgery, Seoul National University Hospital, Seoul, Republic of Korea; ^3^ Department of Obstetrics and Gynecology, Seoul National University College of Medicine, Seoul, Republic of Korea; ^4^ Department of Thoracic and Cardiovascular Surgery, Seoul National University College of Medicine, Seoul, Republic of Korea

**Keywords:** supradiaphragmatic lymphadenectomy, stage IVB ovarian cancer, thoracic lymph node metastasis, residual tumors, overall survival

## Abstract

**Introduction:**

To evaluate the survival impact of supradiaphragmatic lymphadenectomy as part of debulking surgery in stage IVB ovarian cancer with thoracic lymph node metastasis (LNM).

**Methods:**

We retrospectively enrolled patients diagnosed with stage IVB ovarian, fallopian or primary peritoneal cancer between 2010 and 2020, carrying cardiophrenic, parasternal, anterior mediastinal or supraclavicular lymph nodes ≥5 mm on axial chest computed tomography. All tumors were classified into the abdominal (abdominal tumors and cardiophrenic lymph nodes) and supradiaphragmatic (parasternal, anterior mediastinal or supraclavicular lymph nodes) categories depending on the area involved. Residual tumors were classified into <5 vs ≥5 mm in the abdominal and supradiaphragmatic areas. Based on the site of recurrence, they were divided into abdominal, supradiaphragmatic and other areas.

**Results:**

A total of 120 patients underwent primary debulking surgery (PDS, n=68) and interval debulking surgery after neoadjuvant chemotherapy (IDS/NAC, n=53). Residual tumors in the supradiaphragmatic area ≥5 mm adversely affected progression-free survival (PFS) and overall survival (OS) with marginal significance after PDS despite the lack of effect on survival after IDS/NAC (adjusted hazard ratios [HRs], 6.478 and 6.370; 95% confidence intervals [CIs], 2.224-18.864 and 0.953-42.598). Further, the size of residual tumors in the abdominal area measuring ≥5 mm diminished OS after IDS/NAC (adjusted HR, 9.330; 95% CIs, 1.386-62.800).

**Conclusion:**

Supradiaphragmatic lymphadenectomy during PDS may improve survival in patients diagnosed with stage IVB ovarian cancer manifesting thoracic LNM. Further, suboptimal debulking surgery in the abdominal area may be associated with poor OS after IDS/NAC.

**Trial registration:**

ClinicalTrials.gov (NCT05005650; https://clinicaltrials.gov/ct2/show/NCT05005650; first registration, 13/08/2021).

Research Registry (Research Registry UIN, researchregistry7366; https://www.researchregistry.com/browse-the-registry#home/?view_2_search=researchregistry7366&view_2_page=1).

## 1 Introduction

Early diagnosis of ovarian cancer is known to be very important, with a five-year survival rate of over 80% expected (1, 2). However, screening using a combination of transvaginal ultrasound and serum CA-125 levels may not reduce death in the general population significantly, and most patients with ovarian cancer are still diagnosed with stage III-IV disease associated with a fatal prognosis (3, 4).

In particular, stage IVB disease with parenchymal metastasis involving liver and extra-abdominal lymph node metastasis (LNM) ranges from 12% to 15% (1). Since patients with stage IVB disease often carry unresectable tumors during primary debulking surgery (PDS), 25-30% of gynecologic oncologists prefer interval debulking surgery after neoadjuvant chemotherapy (IDS/NAC) due to similar survival benefit but lower rates of complication (5, 6).

However, thoracic LNM, involving cardiophrenic, mediastinal or supraclavicular LNM, is sometimes resectable in collaboration with thoracic surgeons. Thus, supradiaphragmatic lymphadenectomy can lead to optimal debulking surgery (ODS) in patients with stage IVB ovarian cancer and thoracic LNM. Despite this surgical feasibility, comparative evidence of survival benefit associated with supradiaphragmatic lymphadenectomy in these patients is unavailable. Thus, we investigated the effect of supradiaphragmatic lymphadenectomy as a component of ODS on survival of patients in stage IVB ovarian cancer along with thoracic LNM.

## 2 Methods

### 2.1 Patient selection

We searched an institutional database of patients with ovarian, fallopian, or primary peritoneal cancers between 2010 and 2020. The study was designed as a retrospective study, which included only patients with epithelial ovarian, fallopian, or primary peritoneal cancers; underwent PDS or IDS/NAC; International Federation of Obstetrics and Gynecology (FIGO) stage IVB disease; transthoracic LNM such as cardiophrenic, parasternal, anterior mediastinal, or supraclavicular LNM. However, we excluded stage IVB patients with posterior LNM, including middle or posterior mediastinal LNM, pleural seeding, and lung parenchymal metastasis. The Institutional Review Board of Seoul National University Hospital approved this study in advance (No. 1908-173-1059, September 3^rd^ 2019), and we waived the patients’ consent because of a retrospective design. Moreover, this study has been registered in the ClinicalTrials.gov (NCT05005650; https://clinicaltrials.gov/ct2/show/NCT05005650) and the Research Registry (Research Registry UIN, researchregistry7366; https://www.researchregistry.com/browse-the-registry#home/?view_2_search=researchregistry7366&view_2_page=1).

In the earlier years of the study period, our institution followed a standardized protocol for the assessment of patients’ eligibility for complete cytoreduction based on CT and PET-CT scans to evaluate the disease extent and feasibility of ODS. In the later years of the study period, refinements were made to our assessment protocol by incorporating multidisciplinary team approaches to achieve maximal cytoreduction, especially for stage IVB patients presenting supradiaphragmatic metastasis.

### 2.2 Surgical procedures

Abdominal tumors were resected using debulking surgery as described in our previous report (7). First, we performed laparotomic staging operations, including hysterectomy, bilateral salpingo-oophorectomy, and pelvic or para-aortic lymphadenectomy. In this study, we removed enlarged lymph nodes selectively during pelvic or para-aortic lymphadenectomy. In addition, we conducted ultra-radical procedures including appendectomy, splenectomy, distal pancreatectomy, superficial liver mass excision or liver wedge resection, portal triad stripping, bowel resection, and anastomosis with or without prophylactic ileostomy in individual cases as needed. We performed *en bloc* pelvic resection or parietal peritonectomy to remove peritoneal metastasis.

Cardiophrenic lymph node dissection was performed trans-abdominally via diaphragmatic incision or trans-thoracically using video-assisted thoracic surgery (VATS) to remove thoracic LNM. Parasternal or anterior mediastinal lymph nodes were dissected via VATS. In particular, *en bloc* excision of internal mammary vessels near the metastatic lymph nodes was performed. Supraclavicular lymph nodes were dissected via 4 *cm* lateral supraclavicular incision. Bilateral approaches were adopted if indicated. ODS was defined as the size of residual tumors in the abdominal or supradiaphragmatic areas <5 mm after debulking surgery, whereas suboptimal debulking surgery (SDS) was defined as the size ≥5 mm.

### 2.3 Data collection

We collected the following clinical and pathologic parameters: age, American Society of Anesthesiology (ASA) score, treatment types such as PDS and IDS/NAC, tumor origin, histology, use of bevacizumab adjuvant chemotherapy, the size of residual tumors and recurrent sites. We further evaluated the extent of surgical resection and surgical outcomes, including operation time, estimated blood loss, hospitalization, and acute grade 3 or 4 complications based on Memorial Sloan Kettering Cancer Center (MSKCC) grading criteria (8). Moreover, we used the modified Surgical Complexity Score (SCS) system by adding distal pancreatectomy, cholecystectomy, portal triad stripping, adrenalectomy, and lymphadenectomy in the cardiophrenic, internal mammary, and supraclavicular regions to evaluate the level of surgical complexity (9). In the modified SCS system, 18 procedures were scored from 1 to 3, and total scores divided all patients into the following three complexity score groups: low, ≤3; intermediate, 4–7; high, ≥8 (**Supplementary Table 1**).

In this study, we divided the surgical resection area into two compartments. The first compartment included the abdominal area, including abdominal tumors and cardiophrenic lymph nodes because cardiophrenic lymph node dissection was performed by either gynecologic oncologists or thoracic surgeons. The second compartment involved the supradiaphragmatic area, including parasternal, anterior mediastinal, or supraclavicular lymph nodes. Enlarged lymph nodes in the supradiaphragmatic area were resected if they were 5 mm or larger on axial chest computed tomography (CT) before surgery according to the criteria specified in previous studies (10, 11). Thus, based on size, the residual tumors in the adnominal and supradiaphragmatic areas were classified into two groups: <5 and ≥5 mm.

Survival outcomes included progression-free survival (PFS) and overall survival (OS). PFS was defined as the time interval from the treatment start date to the recurrence or last follow-up date. OS was defined as the duration from the treatment start date to cancer-related death or last follow-up date. Further, we investigated the recurrence pattern in the abdominal, supradiaphragmatic, and other areas.

### 2.4 Statistical analysis

The primary outcome of this study is survival outcomes of PFS in patients according to the size of residual disease in the supradiaphragmatic area. The secondary outcome include OS, subgroup analysis in high-grade serous type, surgical outcome, and recurrent sites. Chi-squared or Fisher’s exact test was used to analyze categorical variables and Student’s T-test for continuous variables. Survival outcomes were determined via Kaplan-Meier method with log-rank or Breslow test and identified independent factors affecting PFS and OS via Cox proportional-hazards regression analysis using hazard ratios (HRs) and 94% confidence intervals (CIs). SPSS software version 25.0 (SPSS Inc., Chicago IL, USA) was used for statistical analysis in this study.

## 3 Result

### 3.1 Patient characteristics

**Figure 1:**
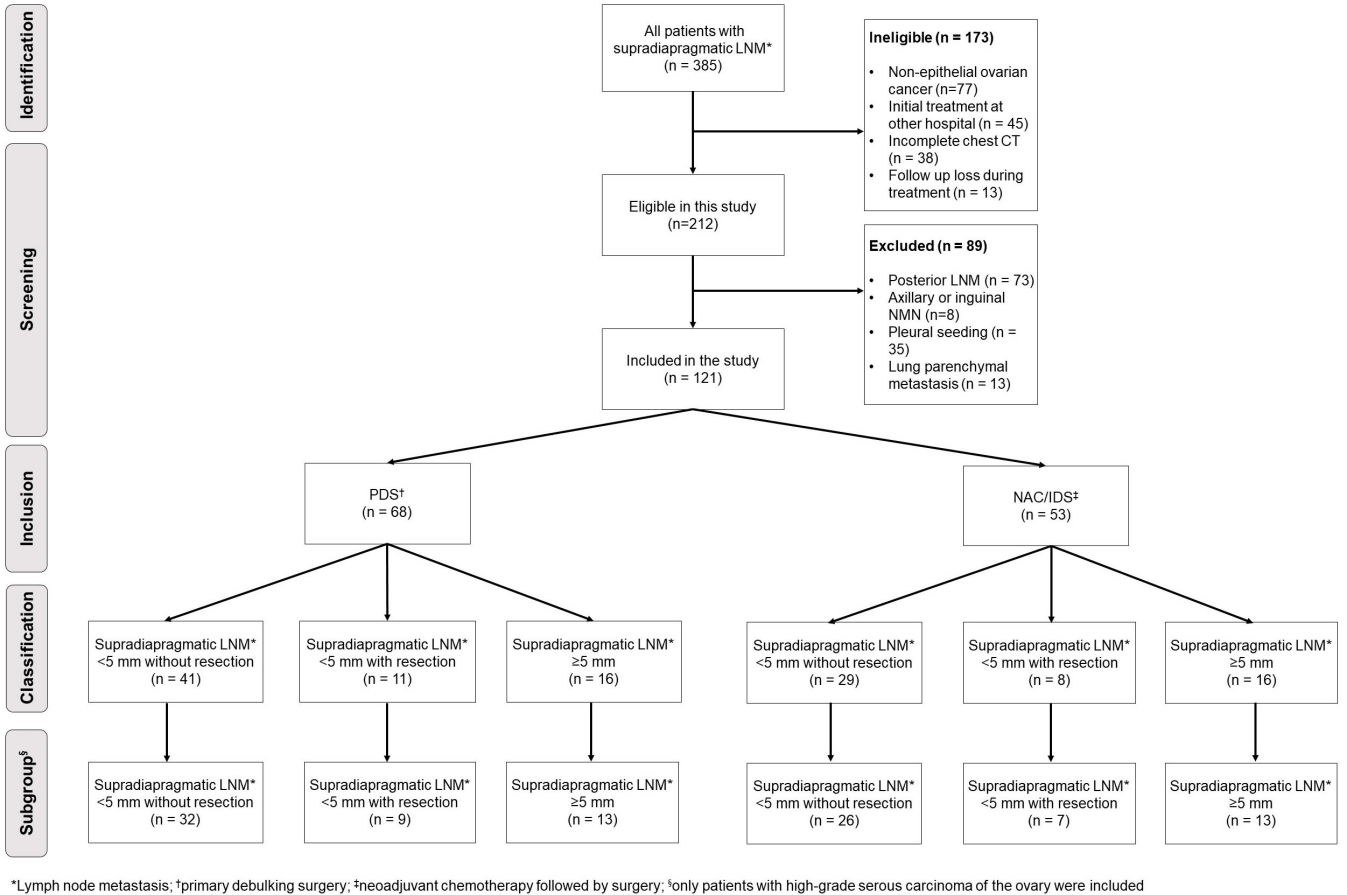
Diagram establishing the study population.

A total of 120 eligible patients were included in our institutional database: 68 (56.2%) patients received PDS, and 53 (43.8%) patients underwent IDS/NAC. Supradiaphragmatic LNM <5 mm without resection, <5 mm with resection and 5 mm were identified in 41 (33.9%), 11 (9.1%) and 16 patients (13.2%) treated with PDS, and 29 (24%), 8 (6.6%) and 16 (13.2%) treated NAC/IDS, respectively. In 100 patients with HGSC of the ovary, supradiaphragmatic LNM <5 mm without resection, <5 mm with resection and 5 mm were identified in 32 (32%), 9 (9%) and 13 patients (13%) treated with PDS, and 26 (26%), 7 (7%) and 13 (13%) treated NAC/IDS, respectively (**Figure 1**).

**Table 1** depicts the clinical and pathological characteristics of all patients. There was no difference in age, ASA score, tumor origin, histology, use of bevacizumab, the size of residual tumors in the abdominal area or the supradiaphragmatic area between patients treated with PDS and those treated with IDS/NAC. Among patients who underwent PDS, 82.4% had histologic type of HGSC, while among patients who underwent IDS, 90.6% had HGSC histologic type.

**Table 1:** Clinico-pathologic characteristics.

Characteristics	PDS (n=68, %)	IDS/NAC (n=53, %)	P value
Age (y)			0.052
<55	37 (54.4)	19 (36.5)	
≥55	31 (45.6)	33 (63.5)	
ASA score			0.628
1	18 (26.5)	12 (22.6)	
2-3	50 (73.5)	41 (77.4)	
Origin			0.884
Ovary	66 (97.1)	51 (96.2)	
Fallopian tube	1 (1.5)	2 (3.8)	
Peritoneum	1 (1.5)	0 (0)	
Histology			0.197
HGSC	56 (82.4)	48 (90.6)	
Non-HGSC	12 (17,6)	5 (9.4)	
Use of bevacizumab			0.333
No	53 (77.9)	45 (84.9)	
Yes	15 (22.1)	8 (15.1)	
Use of First line PARPi			0.409
No	65 (95.6)	52 (98.1)	
Yes	3 (4.4)	1 (1.9)	
*BRCA* status			0.456
Wild type	30 (44.1)	16 (30.2)	
*BRCA1*	9 (13.2)	10 (18.9)	
*BRCA2*	6 (8.8)	5 (9.4)	
Not done	23 (33.8)	22 (41.5)	
The size of residual tumors in the abdominal area*			0.491
<5 mm	40 (58.8)	31 (58.5)	
5 – 10 mm	2 (2.9)	4 (7.5)	
≥10 mm	26 (38.2)	18 (34)	
The size of residual tumors in the supradiaphragmatic area^†^			0.711
<5 mm without resection	41 (60.3)	29 (54.7)	
<5 mm after resection	11 (16.2)	8 (15.1)	
≥5 mm	16 (23.5)	16 (30.2)	

ASA, American Society of Anesthesiology; HGSC, high-grade serous carcinoma; IDS/NAC, interval debulking surgery after neoadjuvant chemotherapy; PARPi, Poly (ADP-ribose) polymerase (PARP) inhibitors; PDS, primary debulking surgery.

* Including abdominal tumors and cardiophrenic lymph nodes.

^†^ Including parasternal, anterior mediastinal, or supraclavicular lymph nodes.

### 3.2 Surgical extents and outcomes

**Supplementary Table 2** shows locations of enlarged and resected lymph nodes, and **Supplementary Table 3** demonstrates the pathologic outcomes of resected lymph nodes in the cardiophrenic, parasternal, anterior mediastinal and supraclavicular regions. There was no difference in the distribution of enlarged and resected lymph nodes and the rate of metastatic lymph nodes among resected cases.

**Table 2** shows the extent of surgical resection during both PDS and IDS/NAC. The two groups did not differ in terms of surgical procedures, including hysterectomy, bilateral salpingo-oophorectomy, pelvic or para-aortic lymphadenectomy, omentectomy, appendectomy, splenectomy, distal pancreatectomy, liver wedge resection, cholecystectomy, portal triad stripping, bowel resection and anastomosis, prophylactic ileostomy, parasternal, anterior mediastinal and supraclavicular lymphadenectomy. However, superficial liver mass excision, diaphragmatic peritonectomy, and cardiophrenic lymphadenectomy were more common in PDS than in IDS/NAC. When we compared the modified SCS between the two groups, high scores ≥8 was more frequent in PDS than in IDS/NAC (77.8% vs 49.1%).

**Table 2:** Extent of surgical resection.

Extent	PDS (n=67, %)	IDS/NAC (n=53, %)	P value
Abdominal area			
Hysterectomy	62 (91.2)	49 (92.5)	1.000
Bilateral salpingo-oophorectomy	68 (100)	53 (100)	–
Pelvic or para-aortic lymphadenectomy	68 (100)	53 (100)	–
Omentectomy	68 (100)	53 (100)	–
Appendectomy	47 (69.1)	31 (58.5)	0.278
Splenectomy	15 (22.1)	8 (15.1)	0.361
Distal pancreatectomy	8 (11.8)	3 (5.7)	0.344
Superficial liver mass excision	17 (25)	2 (3.8)	0.002
Liver wedge resection	5 (7.4)	2 (3.8)	0.403
Cholecystectomy	8 (11.8)	3 (5.7)	0.344
Portal triad stripping	7 (10.3)	3 (5.7)	0.358
Diaphragmatic peritonectomy	40 (58.8)	18 (34)	0.007
Pelvic peritonectomy	41 (60.3)	24 (45.3)	0.100
Small bowel resection and anastomosis	5 (7.4)	3 (5.7)	1.000
Large bowel resection and anastomosis	27 (39.7)	15 (28.3)	0.191
Prophylactic ileostomy	5 (7.4)	1 (1.9)	0.229
Prophylactic chest tube insertion	22 (32.4)	14 (26.4)	0.446
Cardiophrenic lymphadenectomy	31 (46.3)	14 (26.4)	0.026
Supradiaphragmatic area			
Parasternal lymphadenectomy	11 (16.2)	6 (11.3)	0.446
Anterior mediastinal lymphadenectomy	2 (2.9)	1 (1.9)	1.000
Supraclavicular lymphadenectomy	2 (2.9)	2 (3.8)	1.000
Modified surgical complexity score			0.004
Low (≤3)	2 (2.9)	5 (9.4)	
Intermediate (4-7)	13 (19.1)	22 (41.5)	
High (≥8)	53 (77.8)	26 (49.1)	

IDS, interval debulking surgery; NAC, neoadjuvant chemotherapy; PDS, primary debulking surgery.

In terms of surgical outcomes, operation time was longer, and the estimated blood loss was higher in PDS than in IDS/NAC. However, hospitalization and acute grade 3 or 4 complications did not differ between the two groups (**Supplementary Table 4**).

### 3.3 Prognostic factors

When we divided all patients according to the criteria for ODS in the abdominal and supradiaphragmatic areas, no use of bevacizumab after PDS and non-high-grade serous carcinoma (non-HGSC) after IDS/NAC were factors affecting poor PFS and OS (adjusted HRs, 4.214 and 11.445; 95% CIs, 1.648-10.772 and 2.498-52.437), whereas the size of residual tumors in the abdominal and supradiaphragmatic areas ≥5 mm after PDS was a factor affecting poor PFS and OS (adjusted HRs, 1.726 and 2.097; 95% CIs, 0.968-3.077 and 0.665-6.615; **Supplementary Tables 5**, **6**).

For clarifying the effect of supradiaphragmatic lymphadenectomy in these patients, we divided the size of residual tumors in the supradiaphragmatic area into the three groups as follows: <5 mm without resection; <5 mm with resection; ≥5 mm. **Supplementary Table 7** shows the comparison of clinicopathologic characteristics according to the size of residual tumors in the diaphragmatic area. There were no differences in age, ASA score and histology among the three groups, whereas bevacizumab was used more commonly in patients treated with PDS who had the size of residual tumors in the supradiaphragmatic area <5 mm after resection (54.5%), and the size of residual tumors in the abdominal area ≥5 mm was more common when the size of residual tumors in the supradiaphragmatic area was 5 mm or more after IDS/NAC (62.5%).

**Figure 2:**
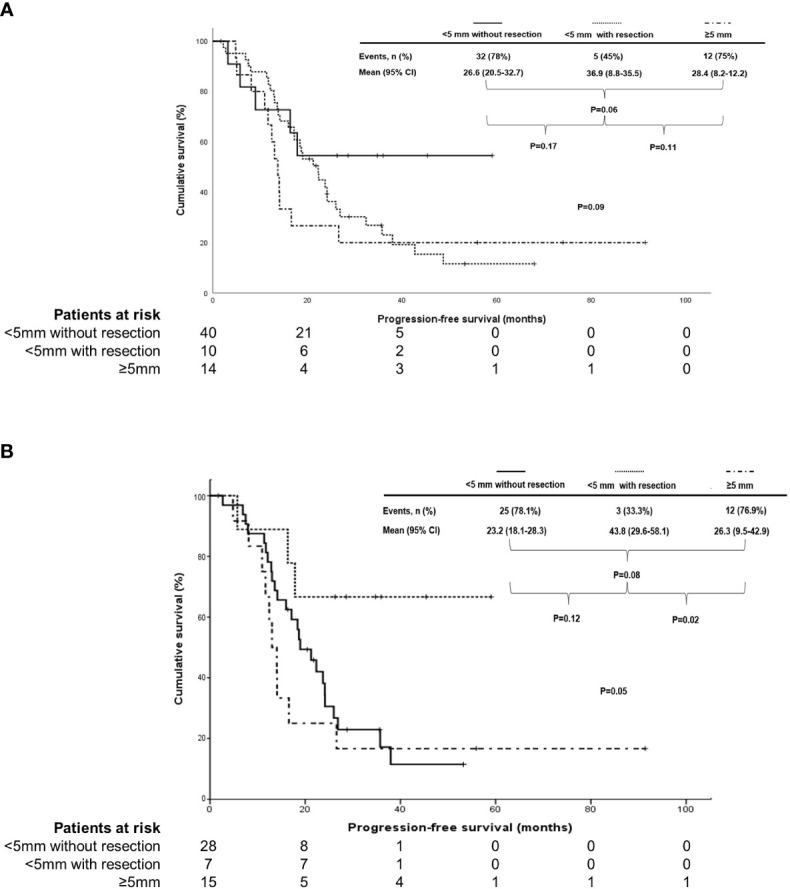
Subgroup analysis in comparison of progression-free survival based on the size of residual tumors in the supradiaphragmatic area (<5 mm without resection vs>5 mm with resection vs≥5 mm) in **(A)** stage IVB patients with thoracic lymph node metastasis who underwent primary debulking surgery (PDS) and in **(B)** those who underwent PDS with high-grade serous carcinoma of the ovary.

In terms of PFS, there were no differences based on the size of residual tumors in the supradiaphragmatic area in patients treated with IDS/NAC and those with HGSC of the ovary (median, 17.1 vs 10 vs 14.7 months; 17.1 vs 22.2 vs 18.2 months in < 5 mm without resection, <5 mm with resection, and ≥5 mm; P = 0.19 and 0.48). However, PFS was different according to the size of residual tumors in the supradiaphragmatic area in patients treated with PDS and those with HGSC of the ovary with marginal significance (mean, 26.6 vs 36.9 vs 28.4 months; 23.2 vs 43.8 vs 26.3 months in <5 mm without resection, <5 mm with resection, and ≥5 mm; P = 0.09 and 0.05; **Figure 2**).

In terms of OS, there was no difference in OS based on the size of residual tumors in the supradiaphragmatic area in patients treated with PDS and those with HGSC of the ovary (mean, 64.8 vs 48.7 vs 103.4 months; 61 vs 47.8 vs 73.5 months in < 5 mm without resection, <5 mm with resection, and ≥5 mm; P=0.94 and 0.88). Moreover, OS was not different according to the size of residual tumors in the supradiaphragmatic area in patients treated with IDS/NAC and those with HGSC of the ovary (mean, 56.8 vs 48.3 vs 74.2 months; 57.1 vs 52 vs 86.8 months in <5 mm without resection, <5 mm with resection, and ≥ 5 mm; P=0.79 and 0.31).

In patients treated with PDS, no use of bevacizumab was an unfavorable factor for PFS (adjusted HR, 7.240; 95% CI, 2.249-23.304), and the size of residual tumors in the supradiaphragmatic area ≥5 mm was an adverse factor for PFS and OS (adjusted HRs, 6.478 and 6.370; 95% CIs, 2.249-23.304 and 2.224-18.864; **Table 3**). When we performed subgroup analyses for only patients with HGSC of the ovary, no use of bevacizumab was also a factor related with decreased PFS (adjusted HR, 7.408; 95% CI, 2.044-26.846), and the size of residual tumors in the supradiaphragmatic area ≥5 mm was also a factor associated with decreased PFS and OS (adjusted HRs, 5.945 and 19.685; 95% CIs, 1.805-19.579 and 1.756-220.660; **Table 4**). However, the size of residual tumors in the supradiaphragmatic area ≥5 mm was not related to survival in those treated with IDS/NAC, whereas the size of residual tumors in the abdominal area was related to decreased OS in patients treated with IDS/NAC and those with HGSC of the ovary (adjusted HRs, 9.330 and 6.209; 95% Cis, 1.386-62.800 and 1.110-34.738; **Supplementary Tables 8**, **9**).

**Table 3:** Factors affecting progression-free and overall survivals in patients treated with primary debulking surgery.

Factors	Univariate			Multivariate		
	HR	95% CI	P value	Adjusted HR	95% CI	P value
Progression-free survival						
Age ≥55 years	0.700	0.395-1.239	0.221	–	–	–
ASA score 2-3	0.860	0.461-1.604	0.636	–	–	–
Non-HGSC	1.010	0.488-2.090	0.979	–	–	–
No use of bevacizumab	3.731	1.473-9.454	0.006	7.240	2.249-23.304	0.001
The size of residual tumors in the abdominal area*						
≥5 mm	0.976	0.541-1.759	0.935	–	–	–
The size of residual tumors in the supradiaphragmatic area^†^						
<5 mm with resection	0.532	0.207-1.369	0.191	–	–	–
≥5 mm	1.301	0.665-2.543	0.442	6.478	2.224-18.864	0.001
Overall survival						
Age ≥55 years	0.711	0.260-1.944	0.507	–	–	–
ASA score 2-3	0.851	0.294-2.463	0.767	–	–	–
Non-HGSC	0.744	0.212-2.827	0.699			
No use of bevacizumab	1.316	0.292-5.932	0.721			
The size of residual tumors in the abdominal area*						
≥5 mm	1.330	0.489-3.621	0.576	–	–	–
The size of residual tumors in the supradiaphragmatic area^†^						
<5 mm with resection	1.219	0.261-5.699	0.801	–	–	–
≥5 mm	0.896	0.278-2.887	0.854	6.370	0.953-42.598	0.056

ASA, American Society of Anesthesiology; CI, confidence interval; HGSC, high-grade serous carcinoma; HR, hazard ratio; PDS, primary debulking surgery.

* Including abdominal tumors and cardiophrenic lymph nodes.

^†^ Including parasternal, anterior mediastinal or supraclavicular lymph nodes.

**Table 4:** Factors affecting progression-free survival in patients with high-grade serous carcinoma of the ovary who underwent primary debulking surgery.

Factors	Univariate			Multivariate		
	HR	95% CI	P value	Adjusted HR	95% CI	P value
Progression-free survival						
Age ≥55 years	0.718	0.367-1.404	0.332	–	–	–
ASA score 2-3	0.752	0.378-1.497	0.418	–	–	–
No use of bevacizumab	5.204	1.824-14.848	0.002	7.408	2.044-26.846	0.002
The size of residual tumors in the abdominal area*						
≥5 mm	1.252	0.662-2.367	0.490	–	–	–
The size of residual tumors in the supradiaphragmatic area^†^						
<5 mm with resection	0.300	0.090-1.999	0.404	–	–	–
≥5 mm	1.371	0.9954-2.874	0.050	5.945	1.805-19.579	0.003

Overall survival						
Age ≥55 years	1.220	0.407-3.655	0.723	–	–	–
ASA score 2-3	0.775	0.230-2.608	0.681	–	–	–
No use of bevacizumab	1.260	0.270-5.873	0.769	–	–	–
The size of residual tumors in the abdominal area*						
≥5 mm	0.863	0.268-2.782	0.805	–	–	–
The size of residual tumors in the supradiaphragmatic area^†^						
<5 mm with resection	1.323	0.274-6.384	0.727	–	–	–
≥5 mm	0.821	0.215-3.144	0.774	19.685	1.756-220.660	0.016

ASA, American Society of Anesthesiology; CI, confidence interval; HGSC, high-grade serous carcinoma; HR, hazard ratio; PDS, primary debulking surgery.

* Including abdominal tumors and cardiophrenic lymph nodes.

^†^ Including parasternal, anterior mediastinal or supraclavicular lymph nodes.

### 3.4 Recurrence patterns

**Supplementary Table 10** depicts specific recurrence sites between PDS and IDS/NAC, which shows no difference in them between the two groups. **Table 5** shows recurrence sites based on the size of residual tumors in the supradiaphragmatic area. As a result, there was no difference in recurrence sites according to the size of residual tumors in the supradiaphragmatic area after PDS and IDS/NAC.

**Table 5:** Recurrence pattern.

Treatment	PDS			IDS		
The size of residual tumors in the supradiaphragmatic area	<5 mm (n=52)	≥5 mm (n=16)	vP value	<5 mm (n=37)	≥5 mm (n=16)	P value
Recurrence sites						
Abdominal area	36 (67.3)	12 (75)	0.458	28 (75.7)	12 (75)	0.607
Pelvic mass	1 (1.9)	1 (6.3)		2 (5.4)	1 (6.3)	
Peritoneal seeding	32 (61.5)	8 (50)		22 (59.5)	8 (50)	
Pelvic node	11 (21.2)	5 (31.3)		6 (16.2)	2 (12.5)	
Para-aortic node	8 (15.4)	6 (37.5)		10 (27)	8 (50)	
Supradiaphragmatic area	5 (9.6)	4 (25)	0.124	4 (10.8)	3 (18.8)	0.353
Parenchymal or other distant sites	6 (11.5)	3 (18.8)	0.355	4 (10.8)	2 (12.5)	0.595
Number of recurrence sites			0.569			0.993	
1	18 (34.6)	6 (67.5)		17 (45.8)	8 (50)	
2	15 (28.8)	3 (18.8)		7 (18.9)	3 (18.8)	
3 or more	4 (7.7)	3 (18.8)		5 (13.5)	2 (12.6)	

IDS/NAC, interval debulking surgery after neoadjuvant chemotherapy; PDS, primary debulking surgery.

## 4 Discussion

VATS has been used to detect unexpected LNM or pulmonary metastasis with surgical feasibility and safety in various types of malignancies (12). Even though VATS has been shown to increase survival by facilitating resection of solitary metastases in some cancers, including colon and pancreatic cancers (13–15), the therapeutic role of resection of multiple metastatic tumors in the intrathoracic area has yet to be reported.

In ovarian cancer, the diagnostic and prognostic roles of VATS are also becoming increasingly important. Based on previous studies, VATS is expected to lead to cancer upstaging and change of management in 41% of patients with advanced ovarian cancer (16). Also, enlarged cardiophrenic lymph nodes may predict upper abdominal disease involvement but still benefit from complete resection of abdominal disease (17, 18). Further, macroscopic intrathoracic disease detected on VATS represents an unfavorable prognostic factor (18, 19). However, the therapeutic effect of tumour resection via VATS is also limited and controversial in ovarian cancer (20). Therefore, this study is the first study of its kind evaluating the therapeutic effect of supradiaphragmatic lymphadenectomy, and the well-known effect of ODS on improved survival may not be apparent in stage IVB ovarian cancer with transthoracic LNM.

In terms of lymphatic drainage, the transthoracic and posterior lymphatic pathways contribute to LNM from the abdomen to the supradiaphragmatic lymph nodes. In particular, lymphatic tumour cells of the abdominal surface of the diaphragm invade the parasternal or anterior mediastinal lymph nodes and further into the supraclavicular lymph nodes via the transthoracic pathway. However, lymphatic tumour cells can diffuse through the diaphragm into the aortic hiatus and the thoracic duct via the posterior lymphatic pathway (21). In this study, we selected only stage IVB patients with transthoracic LNM because LNM via the transthoracic pathway is dominant, and lymphadenectomy by VATS is feasible, compared with LNM via posterior lymphatic pathway, which can lead to ODS in these patients (21–23).

In this study, we defined ODS as the size of residual tumor <5 mm in the abdominal and supradiaphragmatic areas due to the two following reasons. First, lymph nodes <5 mm in the supradiaphragmatic area are not resected with VATS generally because it is difficult to localize them (24). Second, cardiophrenic, parasternal and anterior mediastinal lymph nodes are not palpable or visually notable during VATS because they are located in the extrapleural space (25). Third, thoracic procedures should be performed within a short time without serious complications not to interrupt subsequent abdominal debulking surgery and not to reduce the delivery rate of adjuvant chemotherapy (26). Thus, we conducted VATS as a minimally invasive procedure instead of thoracotomy.

As a result, the rate of ODS was similar between PDS and IDS/NAC in the abdominal and supradiaphragmatic areas, whereas operation time was longer, estimated blood loss was more, and the modified SCS was higher in PDS than in IDS/NAC. However, there was no difference in the rate of grade 3 or 4 complications between the two groups. It means that recovery speed after surgery was similar between the two treatments, which could be supported by the finding of no difference in hospitalization between the two groups. In particular, there was no serious complication related to VATS, requiring subsequent intervention in this study.

In terms of survival, ODS in the supradiaphragmatic area improved PFS and OS, especially in patients undergoing PDS. These findings were also observed in those with HGSC of the ovary after PDS. Even though the results of Lymphadenectomy in Ovarian Neoplasms (LION) included stage IIB-IV disease, and thereby did not reveal the effect of lymphadenectomy in stage IVB disease definitely, it emphasizes that the standard procedure may be to remove only suspicious lymph nodes in a case where complete resection may be reached (27). In this study, we also found that PFS could be improved if you could resect tumors ≥5 mm in the supradiaphragmatic area during PDS, suggesting that supradiaphragmatic lymphadenectomy may be important during ODS for complete resection in stage IVB disease with thoracic LNM.

However, the therapeutic effect of supradiaphragmatic lymphadenectomy was not apparent in patients who underwent IDS/NAC, suggesting that NAC has the potential to decrease the size of enlarged lymph nodes but does not eliminate hidden LNMs, which cannot be resected due to size less than 5 mm on axial chest CT before IDS. This hypothesis is supported by previous studies where radiologic lymph node status after NAC differed from pathologic lymph node status and did not affect survival in advanced ovarian cancer (28, 29).

As the other prognostic factors, no use of bevacizumab decreased PFS after PDS, suggesting that these patients should be included in a high-risk group requiring bevacizumab therapy to decrease the risk of disease recurrence (30). Furthermore, the study findings suggest that SDS in the abdominal area remained associated with poor OS after IDS/NAC. Interestingly, no significant impact on OS was observed after PDS. Although ODS is determined by the size of residual tumors, it is clearly distinct from maximal debulking surgery, defined as the removal of resectable lesions if possible. Previous studies have shown that SDS may reflect a lack of effort to perform maximal debulking surgery, leading to poor prognosis in stage IIIC to IVB disease (31–33). Conversely, the attempt to reduce tumour burden via maximal debulking surgery can be expected to increase OS in stage IVB patients with transthoracic LNM during IDS/NAC. Furthermore, tumors that did not respond well to the NAC and remained after surgery may have a more aggressive biology, leading to a greater negative impact on overall survival.

However, this study has some limitations. First, a retrospective study design is associated with an inherent bias for evaluating the effect of supradiaphragmatic lymphadenectomy. Second, the small number of stage IVB patients with only supradiaphragmatic LNM act as a bias to evaluate the surgical effect. When we consider that the number of stage IVB patients with only supradiaphragmatic LNM is relatively small, and the efficacy of thoracic surgery for these patients has not been reported yet, so there are various positions on surgical resection, which makes it difficult to conduct a multi-center retrospective study, large-scale prospective studies are essential to validate these findings. Third, we excluded patients with stage IVB harboring supradiaphragmatic LNM via the posterior lymphatic pathway because it is relatively rare, and the surgical resection is not facilitated by VATS. Fourth, only a small subset of patients received first-line PARP inhibitors, and a significant number of patients did not undergo genetic testing, conducting a meaningful subgroup analysis based on maintenance therapies and BRCA status becomes challenging. Fifth, modifications to our assessment protocol may have influenced the outcomes observed in our study. It is essential to recognize the evolving nature of advanced ovarian cancer surgical management during the specified time interval. Future research should aim to investigate the impact of these changes on patient outcomes. Sixth, it is important to note that including the cardiophrenic area within the abdominal region for analysis could introduce a potential bias, and therefore, the interpretation of our findings should be approached with caution.

In conclusion, the findings of this study highlight the potential therapeutic benefit of supradiaphragmatic lymphadenectomy during PDS in improving PFS and OS of stage IVB ovarian cancer patients with thoracic LNM. It is important to consider that the impact of supradiaphragmatic lymphadenectomy may diminish during IDS/NAC. Furthermore, SDS in the abdominal area remains associated with poor OS after IDS/NAC. These results emphasize the significance of PDS with no residual tumor as the optimal approach for these patients and highlight the importance of considering these factors in clinical practice and treatment decision-making. Further studies are warranted to validate these findings and optimize the management strategies for stage IVB ovarian cancer patients with thoracic LNM.

## Data availability statement

The raw data supporting the conclusions of this article will be made available by the authors, without undue reservation.

## Ethics statement

The studies involving human participants were reviewed and approved by Seoul National University Hospital Institutional Review Board (1908-173-1059). Written informed consent was not provided because this study poses a low risk to the subjects and utilizes only retrospective medical records, and the Seoul National University Hospital Institutional Review Board has waived the required written consent from the subjects.

## Author contributions

SP and HK conceived and designed this study. SJP, SP and HK collected and analyzed the data. SP, KN, ML, IP, HC, CK, J-WK, NP, Y-TK, YS, SP and HK interpreted the data. SJP, SP, and HK drafted this article. All the authors finally approved the submitted version.
