# Supplementary material for: Impact of supradiaphragmatic lymphadenectomy on the survival of patients in stage IVB ovarian cancer with thoracic lymph node metastasis
Source: Front Oncol. 2023 Aug 10;13:1203127. doi: 10.3389/fonc.2023.1203127 (PMC10448516; doi:10.3389/fonc.2023.1203127)
Supplement: Supplementary file 1 [file DataSheet_1.docx]

Supplementary Material

Impact of supradiaphragmatic lymphadenectomy on the survival of patients in stage IVB ovarian cancer with thoracic lymph node metastasis

Soo Jin Park^1^, Kwon Joong Na^2^, Maria Lee^1.3^, In Kyu Park^2,4^, Hyun Hoon Chung^1,3^, Chang Hyun Kang^2,4^, Jae-Weon Kim^1,3^, Noh Hyun Park^1,3^, Young-Tae Kim^2,4^, Yong Sang Song^1,3^, Samina Park^2,4, *^, Hee Seung Kim^1,3, *^

^1^Department of Obstetrics and Gynecology, Seoul National University Hospital, Seoul 03080, Republic of Korea

^2^Department of Thoracic and Cardiovascular Surgery, Seoul National University Hospital, Seoul 03080, Republic of Korea.

^3^Department of Obstetrics and Gynecology, Seoul National University College of Medicine, Seoul 03080, Republic of Korea

^4^Department of Thoracic and Cardiovascular Surgery, Seoul National University College of Medicine, Seoul 03080, Republic of Korea.

*** Correspondence:**Samina Park, MD

Department of Thoracic and Cardiovascular Surgery, Seoul National University College of Medicine, 101, Daehak-ro Jongno-gu, Seoul 03080, Republic of Korea

Tel: +82-2-2072-2345; E-mail: saminapark1203@gmail.com

Hee Seung Kim, MD, PhD

Department of Obstetrics and Gynecology, Seoul National University College of Medicine, 101, Daehak-ro Jongno-gu, Seoul 03080, Republic of Korea

Tel: +82-2-2072-4863; E-mail: [bboddi0311@snu.ac.kr](mailto:bboddi0311@snu.ac.kr)

# Supplementary table 1. Modified Surgical Complexity Score (SCS) System

| Procedures | Scores |
| --- | --- |
| Hysterectomy with salpingo-oophorectomy | 1 |
| Pelvic or para-aortic lymphadenectomy | 1 |
| Omentectomy | 1 |
| Appendectomy | 1 |
| Splenectomy | 2 |
| Distal pancreatectomy | 2 |
| Superficial liver mass excision | 2 |
| Liver wedge resection | 2 |
| Cholecystectomy | 2 |
| Portal triad stripping | 3 |
| Diaphragmatic peritonectomy | 2 |
| Pelvic peritonectomy | 1 |
| Small bowel resection and anastomosis | 1 |
| Large bowel resection and anastomosis | 3 |
| Prophylactic ileostomy | 1 |
| Cardiophrenic lymphadenectomy | 2 |
| Parasternal or anterior mediastinal lymphadenectomy | 2 |
| Supraclavicular lymphadenectomy | 2 |
| Complexity score groups | Modified SCS |
| Low | ≤3 |
| Intermediate | 4–7 |
| High | ≥8 |

# Supplementary table 2. Locations of enlarged and resected lymph nodes in the cardiophrenic, parasternal, anterior mediastinal and supraclavicular regions

| Location | PDS (n=68, %) | IDS/NAC (n=53, %) | P value |
| --- | --- | --- | --- |
| *Enlarge lymph nodes* |  |  |  |
| Cardiophrenic | | | 0.560 |
| No | 10 (14.7) | 6 (11.3) |  |
| Left | 6 (8.8) | 2 (3.8) |  |
| Right | 42 (61.8) | 34 (64.2) |  |
| Both | 10 (14.7) | 11 (52.4) |  |
| Anterior mediastinal |  |  | 0.593 |
| No | 0 (0) | 0 (0) |  |
| Left | 0 (0) | 0 (0) |  |
| Right | 2 (2.9) | 1 (1.9) |  |
| Both | 66 (97.1) | 52 (98.1) |  |
| Parasternal |  |  | 0.473 |
| No | 52 (76.5) | 34 (64.2) |  |
| Left | 3 (4.4) | 5 (9.4) |  |
| Right | 6 (8.8) | 6 (11.3) |  |
| Both | 7 (10.3) | 8 (15.1) |  |
| Supraclavicular |  |  | 0.067 |
| No | 57 (83.8) | 36 (67.9) |  |
| Left | 10 (14.7) | 12 (22.6) |  |
| Right | 1 (1.5) | 1 (1.9) |  |
| Both | 0 (0) | 4 (7.5) |  |
| *Resected lymph nodes* |  |  |  |
| Cardiophrenic |  |  | 0.035 |
| No | 37 (48.7) | 39 (73.6) |  |
| Left | 2 (2.9) | 0 (0) |  |
| Right | 22 (32.4) | 12 (22.6) |  |
| Both | 7 (10.3) | 2 (3.8) |  |
| Anterior mediastinal |  |  | 0.593 |
| No | 0 (0) | 0 (0) |  |
| Left | 0 (0) | 0 (0) |  |
| Right | 2 (2.9) | 1 (1.9) |  |
| Both | 0 (0) | 0 (0) |  |
| Parasternal |  |  | 0.587 |
| No | 57 (83.8) | 47 (88.7) |  |
| Left | 3 (4.4) | 1 (1.9) |  |
| Right | 4 (5.9) | 4 (7.5) |  |
| Both | 4 (5.9) | 1 (1.9) |  |
| Supraclavicular |  |  | 0.591 |
| No | 0 (0) | 0 (0) |  |
| Left | 2 (2.9) | 2 (3.8) |  |
| Right | 0 (0) | 0 (0) |  |
| Both | 0 (0) | 0 (0) |  |

Abbreviations: IDS, interval debulking surgery; NAC, neoadjuvant chemotherapy; PDS, primary debulking surgery.

# Supplementary table 3. The pathologic outcomes of resected lymph nodes in the cardiophrenic, parasternal, anterior mediastinal and supraclavicular regions

| Location | PDS (n=68) | IDS/NAC (n=53) | P value |
| --- | --- | --- | --- |
| **Cardiophrenic** | | |  |
| No. of resected LN (median, [min-max]) | 3 (1, 16) | 3 (1, 8) | 0.745 |
| No. of metastatic LN (median, [min-max]) | 2 (1, 15) | 3 (1, 4) | 0.673 |
| Metastatic/resected cases (%) | 25 / 31 (80.6) | 9 / 14 (64.3) | 0.208 |
| **Anterior mediastinal** |  |  |  |
| No. of resected LN (median, [min-max]) | 1 (1, 1) | 1 (1, 1) | 1.000 |
| No. of metastatic LN (median, [min-max]) | 1 (1, 1) | 1 (1, 1) | 1.000 |
| Metastatic/resected cases (%) | 2 / 2 (100) | 1 / 1 (100) | - |
| **Parasternal** |  |  |  |
| No. of resected LN (median, [min-max]) | 1 (1, 6) | 3 (1, 5) | 0.392 |
| No. of metastatic LN (median, [min-max]) | 1 (1, 6) | 2.5 (1, 3) | 0.489 |
| Metastatic/resected cases (%) | 6 / 11 (54.5) | 4 / 6 (66.7) | 0.516 |
| **Supraclavicular** |  |  |  |
| No. of resected LN (median, [min-max]) | 5.5 (1, 10) | 10 (10, 10) | 0.439 |
| No. of metastatic LN (median, [min-max]) | 11.5 (5, 18) | 0 (0, 0) | - |
| Metastatic/resected cases (%) | 1 / 2 (50) | 0 / 2 (0) | 0.500 |

Abbreviations: IDS, interval debulking surgery; NAC, neoadjuvant chemotherapy; PDS, primary debulking surgery.

# Supplementary table 4. Surgical outcomes

| Characteristics | PDS (n=67, %) | IDS/NAC (n=53, %) | P value |
| --- | --- | --- | --- |
| Operation time (min)* | 390.2 ± 188.1 | 319.1 ± 137.5 | 0.022 |
| Estimated blood loss (ml)* | 1785.9 ± 1550.2 | 1086.9 ± 932.5 | 0.004 |
| Hospitalization (d)* | 13.9 ± 6.2 | 13.2 ± 5.0 | 0.503 |
| Acute grade 3-4 complications^†^ | | | |
| Gastrointestinal | 5 (7.4) | 3 (5.7) | 1.000 |
| Thromboembolic | 0 (0) | 1 (1.9) | 0.438 |
| Surgical site Infection | 5 (7.4) | 4 (7.5) | 1.000 |

Abbreviations: IDS/NAC, interval debulking surgery after neoadjuvant chemotherapy; PDS, primary debulking surgery.

*All values are depicted with mean ± standard deviation.

^†^Grade 3-4 complications developed within 30 days after surgery based on the Memorial Sloan Kettering Cancer Center (MSKCC) grading criteria.

# Supplementary table 5. Factors affecting progression-free survival according to the size of residual tumors in the abdominal and supradiaphragmatic areas

| Factors | Univariate | | | Multivariate | | |
| --- | --- | --- | --- | --- | --- | --- |
|  | HR | 95% CI | P value | Adjusted HR | 95% CI | P value |
| *PDS* |  |  |  |  |  |  |
| Age ≥55 years | 0.700 | 0.395-1.239 | 0.221 | – | – | – |
| ASA score 2-3 | 0.860 | 0.461-1.604 | 0.636 | – | – | – |
| Non-HGSC | 1.010 | 0.488-2.090 | 0.979 | – | – | – |
| No use of bevacizumab | 3.731 | 1.473-9.454 | 0.006 | 4.214 | 1.648-10.772 | 0.003 |
| The size of residual tumors in the abdominal* and supradiaphragmatic^†^ areas | | | | | | |
| ≥5 mm | 1.702 | 0.955-3.035 | 0.071 | 1.726 | 0.968-3.077 | 0.064 |
| *IDS/NAC* | | | | | | |
| Age ≥55 years | 1.985 | 1.021-3.858 | 0.043 | – | – | – |
| ASA score 2-3 | 0.877 | 0.437-1.757 | 0.710 | – | – | – |
| Non-HGSC | 1.990 | 0.702-5.639 | 0.195 | – | – | – |
| No use of bevacizumab | 1.509 | 0.591-3.857 | 0.390 | – | – | – |
| The size of residual tumors in the abdominal* and supradiaphragmatic^†^ areas | | | | | | |
| ≥5 mm | 0.998 | 0.547-1.825 | 0.998 | – | – | – |

Abbreviations: ASA, American Society of Anesthesiology; CI, confidence interval; HGSC, high-grade serous carcinoma; HR, hazard ratio; IDS/NAC, interval debulking surgery after neoadjuvant chemotherapy; PDS, primary debulking surgery.

*Including abdominal tumors and cardiophrenic lymph nodes.

^†^Including parasternal, anterior mediastinal or supraclavicular lymph nodes.

# Supplementary table 6. Factors affecting overall survival according to the size of residual tumors in the abdominal and supradiaphragmatic areas

| Factors | Univariate | | | Multivariate | | |
| --- | --- | --- | --- | --- | --- | --- |
|  | HR | 95% CI | P value | Adjusted HR | 95% CI | P value |
| *PDS* |  |  |  |  |  |  |
| Age ≥55 years | 0.711 | 0.260-1.944 | 0.507 | – | – | – |
| ASA score 2-3 | 0.851 | 0.294-2.463 | 0.767 | – | – | – |
| Non-HGSC | 0.744 | 0.212-2.827 | 0.699 | – | – | – |
| No use of bevacizumab | 1.316 | 0.292-5.932 | 0.721 |  |  |  |
| The size of residual tumors in the abdominal* and supradiaphragmatic^†^ areas | | | | | | |
| ≥5 mm | 1.524 | 0.540-4.304 | 0.426 | 2.097 | 0.665-6.615 | 0.077 |
| *IDS/NAC* | | | | | | |
| Age ≥55 years | 0.573 | 0.200-1.640 | 0.299 | – | – | – |
| ASA score 2-3 | 0.534 | 0.178-1.604 | 0.264 | – | – | – |
| Non-HGSC | 7.170 | 1.869-27.498 | 0.004 | 11.445 | 2.498-52.437 | 0.002 |
| No use of bevacizumab | 0.644 | 0.136-3.045 | 0.579 | – | – | – |
| The size of residual tumors in the abdominal* and supradiaphragmatic^†^ areas | | | | | | |
| ≥5 mm | 0.744 | 0.223-2.484 | 0.631 | – | – | – |

Abbreviations: ASA, American Society of Anesthesiology; CI, confidence interval; HGSC, high-grade serous carcinoma; HR, hazard ratio; IDS/NAC, interval debulking surgery after neoadjuvant chemotherapy; PDS, primary debulking surgery.

*Including abdominal tumors and cardiophrenic lymph nodes.

^†^Including parasternal, anterior mediastinal or supraclavicular lymph nodes.

# Supplementary table 7. Comparison of clinicopathologic characteristics according to the size of residual tumors in the supradiaphragmatic area*

| Characteristics | PDS | | | P value | IDS/NAC | | | P value |
| --- | --- | --- | --- | --- | --- | --- | --- | --- |
|  | <5 mm without resection (n=41, %) | <5 mm with resection (n=11, %) | ≥5 mm (n=16, %) |  | <5 mm without resection (n=29, %) | <5 mm with resection (n=8, %) | ≥5 mm (n=16. %) |  |
| Age |  |  |  | 0.917 |  |  |  | 0.466 |
| ≥55 | 18 (43.9) | 5 (45.5) | 8 (50) |  | 19 (65.5) | 3 (37.5) | 11 (68.8) |  |
| ASA score |  |  |  | 0.179 |  |  |  | 0.757 |
| 2-3 | 27 (65.9) | 10 (90.9) | 13 (81.3) |  | 22 (75.9) | 7 (87.5) | 12 (75) |  |
| Histology |  |  |  | 0.988 |  |  |  | 0.262 |
| Non-HGSC | 7 (17.1) | 2 (22.2) | 3 (18.8) |  | 2 (6.9) | 0 (0) | 3 (18.8) |  |
| Use of bevacizumab |  |  |  | 0.010 |  |  |  | 0.429 |
| Yes | 5 (12.2) | 6 (54.5) | 4 (25) |  | 5 (17.2) | 2 (25) | 1 (6.3) |  |
| The size of residual tumors in the abdominal area† | | |  | 0.105 |  |  |  | 0.044 |
| ≥5 mm | 21 (51.2) | 2 (18.2) | 9 (56.3) |  | 11 (37.9) | 1 (12.5) | 10 (62.5) |  |

Abbreviation: ASA, American Society of Anesthesiology; HGSC, high-grade serous carcinoma; IDS/NAC, interval debulking surgery after neoadjuvant chemotherapy; PDS, primary debulking surgery.

*Including parasternal, anterior mediastinal, or supraclavicular lymph nodes.

†Including abdominal tumors and cardiophrenic lymph nodes.

# Supplementary table 8. Factors affecting progression-free survival and overall survival in all patients treated with interval debulking surgery after neoadjuvant chemotherapy

| Factors | Univariate | | | Multivariate | | |
| --- | --- | --- | --- | --- | --- | --- |
|  | HR | 95% CI | P value | Adjusted HR | 95% CI | P value |
| *Progression-free survival* |  |  |  |  |  |  |
| Age ≥55 years | 1.985 | 1.021-3.858 | 0.043 | – | – | – |
| ASA score 2-3 | 0.877 | 0.437-1.757 | 0.710 | – | – | – |
| Non-HGSC | 1.990 | 0.702-5.639 | 0.195 | – | – | – |
| No use of bevacizumab | 1.509 | 0.591-3.857 | 0.390 |  |  |  |
| The size of residual tumors in the abdominal area* | | | | | | |
| ≥5 mm | 1.020 | 0.547-1.902 | 0.949 | – | – | – |
| The size of residual tumors in the supradiaphragmatic area^†^ | | | | | | |
| <5 mm with resection | 0.679 | 0.270-1.704 | 0.409 | – | – | – |
| ≥5 mm | 0.977 | 0.499-1.910 | 0.945 | – | – | – |
| *Overall survival* | | | | | | |
| Age ≥55 years | 0.573 | 0.200-1.640 | 0.299 | – | – | – |
| ASA score 2-3 | 0.534 | 0.178-1.604 | 0.264 | – | – | – |
| Non-HGSC | 7.170 | 1.869-27.498 | 0.004 | 11.725 | 1.869-73.540 | 0.009 |
| No use of bevacizumab | 0.644 | 0.136-3.045 | 0.579 | – | – | – |
| The size of residual tumors in the abdominal area* | | | | | | |
| ≥5 mm | 1.647 | 0.575-4.717 | 1.647 | 9.330 | 1.386-62.800 | 0.022 |
| The size of residual tumors in the supradiaphragmatic area^†^ | | | | | | |
| <5 mm with resection | 0.625 | 0.130-3.002 | 0.557 | – | – | – |
| ≥5 mm | 0.744 | 0.223-2.484 | 0.631 | – | – | – |

Abbreviations: ASA, American Society of Anesthesiology; CI, confidence interval; HGSC, high-grade serous carcinoma; HR, hazard ratio; IDS/NAC, interval debulking surgery after neoadjuvant chemotherapy.

*Including abdominal tumors and cardiophrenic lymph nodes.

^†^Including parasternal, anterior mediastinal or supraclavicular lymph nodes.

# Supplementary table 9. Factors affecting progression-free survival and overall survival in patients treated with interval debulking surgery with high-grade serous histologic type

| Factors | Univariate | | | Multivariate | | |
| --- | --- | --- | --- | --- | --- | --- |
|  | HR | 95% CI | P value | Adjusted HR | 95% CI | P value |
| *Progression-free survival* |  |  |  |  |  |  |
| Age ≥55 years | 1.348 | 0.346-5.243 | 0.667 | – | – | – |
| ASA score 2-3 | 0.420 | 0.117-1.508 | 0.184 | – | – | – |
| No use of bevacizumab | 0.342 | 0.062-1.884 | 0.218 | 12.162 | 1.164-127.105 | 0.037 |
| The size of residual tumors in the abdominal area* | | | | | | |
| ≥5 mm | 1.466 | 0.422-5.098 | 0.547 | 6.209 | 1.110-34.738 | 0.038 |
| The size of residual tumors in the supradiaphragmatic area^†^ | | | | | | |
| <5 mm with resection | 0.324 | 0.039-2.711 | 0.299 | – | – | – |
| ≥5 mm | 0.379 | 0.078-1.838 | 0.229 | – | – | – |
| *Overall survival* | | | | | | |
| Age ≥55 years | 1.348 | 0.346-5.243 | 0.667 | – | – | – |
| ASA score 2-3 | 0.420 | 0.117-1.508 | 0.184 | – | – | – |
| No use of bevacizumab | 0.342 | 0.062-1.884 | 0.218 | 12.162 | 1.164-127.105 | 0.037 |
| The size of residual tumors in the abdominal area* | | | | | | |
| ≥5 mm | 1.466 | 0.422-5.098 | 0.547 | 6.209 | 1.110-34.738 | 0.038 |
| The size of residual tumors in the supradiaphragmatic area^†^ | | | | | | |
| <5 mm with resection | 0.324 | 0.039-2.711 | 0.299 | – | – | – |
| ≥5 mm | 0.379 | 0.078-1.838 | 0.229 | – | – | – |

Abbreviations: ASA, American Society of Anesthesiology; CI, confidence interval; HGSC, high-grade serous carcinoma; HR, hazard ratio; IDS/NAC, interval debulking surgery after neoadjuvant chemotherapy.

*Including abdominal tumors and cardiophrenic lymph nodes.

^†^Including parasternal, anterior mediastinal or supraclavicular lymph nodes.

# Supplementary table 10. Specific recurrence sites

| Location | PDS (n=68, %) | IDS/NAC (n=53, %) | P value |
| --- | --- | --- | --- |
| Pelvic cavity | 2 (2.9) | 3 (5.7) | 0.383 |
| Diffuse peritoneum | 40 (58.8) | 30 (56.6) | 0.422 |
| Pelvic lymph nodes | 8 (11.8) | 5 (9.4) | 0.458 |
| Para-aortic lymph nodes | 14 (20.6) | 18 (34) | 0.074 |
| Pleura | 2 (2.9) | 3 (5.7) | 0.383 |
| Neck or axillary lymph nodes | 3 (4.4) | 2 (3.8) | 0.617 |
| Bone | 1 (1.5) | 0 | 0.562 |
| Liver parenchyme | 2 (2.9) | 1 (1.9) | 0.593 |
| Cardiophrenic lymph nodes | 16 (23.5) | 9 (17) | 0.257 |
| Parasternal lymph nodes | 2 (2.9) | 4 (7.5) | 0.230 |
| Supraclavicular lymph nodes | 8 (11.8) | 4 (7.5) | 0.325 |
| Middle mediastinal lymph nodes | 1 (1.5) | 1 (1.9) | 0.686 |
| Inguinal lymph nodes | 2 (2.9) | 1 (1.9) | 0.593 |

Abbreviations: IDS, interval debulking surgery; NAC, neoadjuvant chemotherapy; PDS, primary debulking surgery.
